# Individual Research Behaviors and Research Funding Acquisition Across Fields and Career Periods: Regression Analysis

**DOI:** 10.2196/98428

**Published:** 2026-07-27

**Authors:** Akiko Hashiguchi, Satoru Takahashi

**Affiliations:** 1 Planning Committee, University of Tsukuba, 1-1-1 Tennodai, Tsukuba, Ibaraki, 305-8575, Japan, 81 298-53-7276

**Keywords:** researcher performance, funding acquisition, Grant-in-Aid for Scientific Research (KAKENHI), research-related behaviors, collaborative research, research agenda reconfiguration

## Abstract

**Background:**

Acquiring external funding for academic research is a crucial element of researcher performance assessment. However, funding opportunities are often concentrated among a limited number of researchers, reinforcing existing structural inequalities. Under these conditions, it is important to clarify how individual researchers can improve their ability to secure external funding through their own actions and thereby build sustainable academic careers. In addition, fair faculty evaluation requires recognizing disciplinary differences in funding acquisition without assuming a normative research environment.

**Objective:**

This study aimed to examine whether research-related behaviors at different career stages contribute to variations in funding acquisition capability across research fields.

**Methods:**

We used funding data from Grant-in-Aid for Scientific Research, Japan’s largest competitive research funding program, and classified 1152 professors according to their lifetime patterns of research funding acquisition. We also collected data on individual research-related behavioral characteristics, including supervisor prestige, authorship position, publication quality, collaborative research structure, and funding acquisition strategies, from publication records and administrative data, constructing a dataset of 804 researchers. By integrating these datasets, we conducted univariate and multivariate regression analyses to examine the factors associated with research funding acquisition.

**Results:**

Field-specific differences were observed in the upper range of funding levels and individual behaviors. High-funding recipients were found only in the field of basic and internal medicine. Univariate logistic regression analyses showed that early-career publication success (basic medicine: odds ratio [OR] 1.48, 95% CI 1.22‐1.79; internal medicine: OR 1.34, 95% CI 1.06‐1.69; and surgery: OR 1.70, 95% CI 1.28‐2.27) and midcareer senior authorship were significantly associated with funding acquisition (basic medicine: OR 5.99, 95% CI 2.63‐13.65; internal medicine: OR 3.17, 95% CI 1.16‐8.70; and surgery: OR 9.79, 95% CI 3.26‐29.43). In addition, supporting specific collaborators (surgery: OR 2.09, 95% CI 1.10‐3.97) and the quality of publications during the first 12 years of a researcher’s career were major determinants of success (surgery: OR 2.22, 95% CI 1.18‐4.17). In multiple linear regression analyses with total funding as the outcome, the adjusted *R*² values were 0.576 for surgery, 0.890 for social medicine, and 0.738 for dentistry, indicating that the models accounted for many behavioral factors associated with funding acquisition. However, the model fit suggests that additional relevant factors may influence funding acquisition in basic medicine, internal medicine, and nursing.

**Conclusions:**

This study identified individual behaviors that influence Grant-in-Aid for Scientific Research funding acquisition across research fields. While organizational and academic structures have a significant impact, personal initiative during researchers’ transition from peripheral to core roles becomes important, emphasizing strong relationship-building skills and outstanding publication records. As effective actions and their timing vary by field, individuals must consider their field characteristics and implement appropriate measures at suitable times.

## Introduction

The increasing emphasis on quantifiable outcomes has normalized metric-driven evaluation in faculty career progression. Publishing productivity and acquisition of external funding are crucial elements in researcher performance assessment [1]. External funding not only enhances a university’s reputation as an external evaluator of researchers’ competence but also holds significant importance for university management under financial constraints [2].

The pronounced Matthew effect, whereby success in securing research funding leads to further success, should be taken into account when evaluating researchers’ performance [3-5]. Since the 2000s, there has been a significant disparity in research funding acquisition, with a Gini coefficient exceeding 0.6, indicating that a disproportionate share of funding is concentrated among a small number of elite researchers [6]. Although total US health research funding has doubled, National Institutes of Health (NIH) Research Project Grants have become increasingly concentrated, with approximately 10% of funding going to a small number of elite researchers (the top 1%) [7]. A researcher’s affiliated institution, past awards, and outstanding publication records create a “halo effect” that influences evaluators’ judgments and, ultimately, determines the success or failure of research grant applications [8]. Previous studies have identified factors influencing funding acquisition, including the principal investigator’s (PI) gender [9], age, academic rank [10], publication quality, international visibility [11], experience in collaborative research [12], and research interdisciplinarity [13]. These factors amplify and entrench initial, minor disparities throughout the course of a researcher’s career. In other words, before researchers are able to lead original research grounded in their own experience, the success of their careers is strongly shaped by structural factors beyond their control.

Research on the “science of science” emphasizes that scientific success is structurally linked to embeddedness in elite academic networks rather than individual excellence [14,15]. The identified characteristics of outstanding researchers have led to policies aimed at fostering excellence, such as enhancing international mobility, promoting team science, and encouraging interdisciplinary collaboration. However, when considering the cumulative advantages, the factors regarded as determinants of scientific success can be understood as reflecting a researcher’s position within organizational and academic structures—specifically, holding senior positions at prestigious universities. Consequently, research performance assessment based on currently available quantitative indicators may have limited value in assessing sustained and incremental academic contributions.

In parallel with growing recognition of persistent structural inequalities in academia, recent studies have increasingly examined the strategies through which researchers sustain their careers under such conditions. According to a study that analyzed the career development process at each stage in detail, researchers strategically exercised agency by adopting and constructing their own professional roles to secure the resources necessary to maximize their achievements as their careers progress [16]. In this context, investigating how the strategies adopted at different career stages contribute to the long-term sustainability of individual funding acquisition could provide insights for sustaining individual careers as well as maintaining the vitality of the research ecosystem [17].

Previous studies on academic success assumed a normative research environment and did not account for field-specific research practices. There is a well-known disparity between the humanities and natural sciences in publication and citation counts. In the medical field, the number of researchers exceeding a given threshold for the *h*-index varies by specialty [18], and the success rate of obtaining research grants as one’s career progresses, particularly the transition from NIH-mentored career development (K) grants to independent investigator-initiated (R) grants, differs by specialty [19]. Disregard for field-specific contexts hinders fair evaluation within medical schools that encompass basic, clinical, and translational research. An accurate understanding of the differences in funding acquisition across fields is essential for valid researcher performance assessment.

Accordingly, this study investigated whether research-related behaviors at different career stages contribute to variations in funding acquisition capability across research fields.

## Methods

### Overview

In Japan, the Grant-in-Aid for Scientific Research (GIA) provides fundamental financial support for academic research in all fields. GIA offers a range of programs, including basic programs classified by the amount awarded, innovation programs that address new challenges, and specially promoted research programs that support outstanding original research and pioneer new areas of study. Funding allocation is based on evaluations of researchers’ past achievements and the originality, scientific significance, feasibility, and soundness of proposed research projects. Therefore, a track record of securing GIA grants directly demonstrates a researcher’s capabilities and future potential. For this reason, the amount of GIA funding received by each national university is a key indicator in university evaluations and is also a major metric for faculty recruitment and promotion.

### Data Source

Researchers in the medical field were extracted from the GIA database [20]. Given that GIA is effectively the only source of public funding scheme in Japan that supports investigator-initiated (bottom-up) research and that a track record of securing such grants is highly valued in recent faculty recruitment, it is reasonable to assume that this database covers nearly all researchers actively engaged in medical research at Japan’s national universities, with the exception of those in the early stages of their careers or those whose primary duties are clinical work.

As many GIA-funded projects span 3 to 4 years, extracting researchers who initiated new projects in a given fiscal year enables us to identify approximately one-third of the population of scholars continuously engaged in GIA-funded research. Annual fluctuations in the amount and distribution of GIA grants reported by the funding agency are minimal; therefore, this sampling method yields a sample that is representative of the population.

In the GIA database, research institutions and implementation periods can be used as search terms to obtain information such as project titles, program categories, grant amounts, and researcher names. Information on the year of degree acquisition was obtained from the CiNii Research database [21] and used as an alternative indicator of researcher age, as exact ages were not publicly available. Each researcher’s productivity and publication quality were calculated from publication histories using data from the SciVal database, which is licensed by the University of Tsukuba, with the Scopus IDs as the search key [22].

### Ethical Considerations

This study did not require ethical approval because it did not involve data collection from human or animal subjects. Researchers’ names and their grant statuses are publicly available. The GIA database, which is managed by the Ministry of Education, Culture, Sports, Science, and Technology in Japan, was created to ensure transparency in public research funding allocations and allow users to search for current research information in Japan. Researchers’ publication statuses are available under the commercial license. The analysis plan was not registered because this study was a secondary analysis of data extracted from databases.

### Samples

We focused on medical school professors to understand how various activities at different career stages influence the success of research proposals over time. We identified 1526 professors as participants from national university medical schools who had secured new GIA projects in 2024 (Table 1). To understand the career stages of these professors at the time they received GIA grants, we analyzed the years in which they obtained their doctoral degrees. This allowed us to identify the doctoral graduation years and institutions of 1157 professors (Table 1). We then used the number of years since degree completion to examine when and through which programs these professors received project grants, thereby identifying patterns in GIA acquisition across career stages.

**Table 1.** Researcher population and their characteristics.

Population	N	Female, n (%)	Field, n (%)					
			Basic medicine	Clinical medicine (internal medicine)	Clinical medicine (surgery)	Social medicine	Nursing	Dentistry
Initial sample (N_0_)	1526	187 (12.25)	424 (27.79)	283 (18.48)	446 (29.23)	105 (6.88)	152 (9.96)	116 (7.60)
Degree matched (N_1_)	1152	87 (7.55)	369 (32.03)	229 (19.88)	338 (29.34)	62 (5.38)	59 (5.12)	95 (8.25)
Publication record retrieved (N_2_)	804	68 (8.46)	240 (29.85)	162 (20.14)	247 (30.72)	48 (5.97)	54 (6.71)	53 (6.59)

We further linked the data for the 1157 professors to behavioral indicators derived from their publication records. As SciVal provides publication data only from 1996 onward, the analysis was restricted to researchers who obtained their doctoral degrees in or after 1996 to capture publication activities from the early stages of their careers. Using SciVal, we successfully identified publication histories for 804 professors. For the calculation of behavioral indicators, we included all publication types rather than restricting the analysis to research articles alone. This approach was intended to capture diverse forms of scholarly contribution and account for the increasing diversity of publication practices accompanying the expansion of interdisciplinary research. Subsequent analyses were therefore conducted using this subset (Table 1).

### Metrics

The metrics used in this study are as follows (Multimedia Appendix 1). For researcher competitiveness, we calculated the total grant amount secured as a PI (total funding) and the total grant amount secured as a PI during the 12 years following the completion of a doctoral degree (funding_0–12y). The latter was included as a lagged variable to mitigate endogeneity.

Individual attributes include the number of years since obtaining a doctoral degree (career length), gender (gender), and the rank of the university where each researcher obtained their doctoral degree (university rank [graduated]) and the university with which they are currently affiliated (university rank [currently affiliated]).

To evaluate the quality of research output and leadership throughout a researcher’s career, we calculated several metrics based on publication data: the total number of publications (total publication counts); the proportion of publications in which the researcher was listed as the first, second, or senior author (% lead-authored publications); and the average Source Normalized Impact per Paper (SNIP) of lead-authored publications (average SNIP). Journal SNIP scores were used as a proxy for research quality rather than paper-level metrics because they demonstrate that a paper meets the journal’s publication standards. These metrics indicate the researcher’s overall scholarly strength.

Furthermore, to clarify research activities and achievements during the initial career stage, we quantified the mentor’s competitiveness (mentor level). On the basis of the organizational structure of GIA projects, we defined a participant’s mentor as the PI who first appointed the participant as a co-investigator in a GIA project. Mentor level was quantified using the largest GIA awarded to that mentor. Additionally, we calculated the number of first-authored publications produced within the first 5 years after degree completion (#first-authored_0–5y) and the total SNIP of those publications (SNIP_0–5y).

To identify participants’ strengths as collaborators and leaders within research networks, we calculated the following two metrics: (1) the ratio of total to unique PIs across projects involving the participant as a project member, reflecting the extent to which a researcher is repeatedly recruited by the same PI, capturing the researcher’s value and reliability as a research collaborator and their access to established research environments (elite reliance); and (2) the ratio of total to unique collaborators across projects secured by the participant as a PI, reflecting the extent to which a researcher repeatedly recruits the same collaborators across their own projects, capturing the ability to build and sustain collaborative research networks (selective mobilization ability). These 2 indicators were conceived based on the findings suggesting that early-career researchers enhance their competitiveness by gaining experience in large-scale projects led by established researchers, while established researchers improve their research outcomes by connecting projects involving diverse participants under a broad conceptual framework [23,24].

Regarding researchers’ funding acquisition strategies, we calculated the number of research projects secured as a PI (# projects); the number of research projects secured as a PI in large-scale research programs (Grant-in-Aid for Specially Promoted Research, Grants-in-Aid for Scientific Research (S), and (A)) (# large-scale projects); and the proportion of research grants acquired as a PI in the category of Challenging Exploratory Research (which, following institutional reforms, includes Grant-in-Aid for Exploratory Research, Challenging Exploratory Research, and Challenging Research [Pioneering] or [Exploratory]) (% challenging research). In this context, engagement in large-scale research programs was interpreted as indicating commitment to a core research agenda, whereas participation in challenging exploratory research was interpreted as indicating engagement with complementary research agendas.

Finally, to examine research performance across different career stages, we divided academic careers into 3 periods according to years since degree completion: early career (0‐12 y), midcareer (13‐24 y), and late career (≥25 y). For each stage, we calculated the average SNIP of publications in which the researcher served as the first, second, or senior author. Indicator names were defined according to author position and career stage. For example, SNIP_first_0–12y denotes the average SNIP of first-authored publications during the early career stage, whereas SNIP_second_13–24y and SNIP_senior_25+y denote the corresponding indicators for second-authored publications during the midcareer stage and senior-authored publications during the late career stage, respectively.

### Statistical Analysis

Agglomerative hierarchical clustering was performed using XLSTAT statistical software (Addinsoft) to group the participants. Univariate logistic regression and multiple linear regression analyses were conducted using XLSTAT to estimate the impact of factors such as career length, university ranking, professional networks, productivity, output quality, leadership in academic writing, and grant acquisition strategies on GIA acquisition.

## Results

### Researcher Types Based on Funding Acquisition Status and Distribution by Field

Table 2 presents the descriptive statistics of the variables used in the analysis. For agglomerative hierarchical clustering, we calculated the total GIA funding acquired throughout each researcher’s career as a PI and funding acquired during the first 12 years. Researchers (N=1152) were grouped based on total funding (indicator: total funding), early career funding (indicator: funding_0–12y), and career duration (indicator: career length). Six phenotype clusters were identified (Table 3). On the basis of the total funding and acquisition patterns (Multimedia Appendix 2), the groups were classified as follows: group 1, established type; group 2, emerging type; group 3, catching-up trajectory type; group 4, plateaued type; group 5, moderate-level stable type; and group 6, low-slope stable type.

**Table 2.** Descriptive statistics.

Metrics	Degree matched (N_1_=1152)				Publication record retrieved (N_2_=804)			
	Minimum	Maximum	Median (IQR)	Mean (SD)	Minimum	Maximum	Median	Mean (SD)
Total funding (million US $^a^)	0.01	16.02	0.35 (0.17‐0.90)	0.99 (1.93)	0.00	7.17	0.27 (0.16‐0.77)	0.83 (0.51)
Funding_0–12y (million US $^a^)	0.00	7.17	0.10 (0.03‐0.20)	0.18 (0.44)	0.01	15.44	0.10 (0.04‐0.20)	0.21 (1.68)
Career length (y)	11.00	48.00	26.00 (21.00‐30.00)	25.55 (5.99)	11.00	29.00	23.00 (20.00‐26.00)	22.76 (4.33)
University rank (currently affiliated)^b^	0.00	1.00	0.00 (0.00‐0.00)	0.08 (0.26)	1.00	7.00	5.00 (2.00‐5.00)	4.20 (1.74)
University rank (graduated)^b^	1.00	7.00	4.00 (2.00‐5.00)	4.18 (1.76)	1.00	7.00	3.00 (2.00‐5.00)	3.25 (1.83)
# projects	**—** ^c^	**—**	**—**	**—**	1.00	37.00	6.00 (4.00‐8.00)	6.64 (4.06)
# large-scale projects	**—**	**—**	**—**	**—**	0.00	6.00	0.00 (0.00‐0.00)	0.16 (0.58)
% challenging research	**—**	**—**	**—**	**—**	0.00	4.00	0.00 (0.00‐0.25)	0.16 (0.33)
Total publication counts	**—**	**—**	**—**	**—**	1.00	901.00	123.50 (72.00‐207.00)	159.46 (129.85)
% lead-authored publications	**—**	**—**	**—**	**—**	0.00	1.64	0.53 (0.40‐0.68)	0.55 (0.22)
Average SNIP^d^	**—**	**—**	**—**	**—**	0.00	3.26	0.78 (0.62‐0.99)	0.83 (0.36)
# first-authored_0–5y	**—**	**—**	**—**	**—**	0.00	49.00	3.00 (1.00‐5.00)	3.56 (4.14)
SNIP_0–5y	**—**	**—**	**—**	**—**	0.00	12.18	1.37 (0.51‐2.05)	1.54 (1.50)
SNIP_first_0–12y	**—**	**—**	**—**	**—**	0.00	13.22	0.67 (0.37‐1.07)	0.84 (0.85)
SNIP_first_13–24y	**—**	**—**	**—**	**—**	0.00	3.83	0.00 (0.00‐0.07)	0.12 (0.32)
SNIP_first_25+y	**—**	**—**	**—**	**—**	0.00	14.59	0.68 (0.23‐1.53)	1.29 (1.86)
SNIP_second_0–12y	**—**	**—**	**—**	**—**	0.00	7.21	1.01 (0.76‐1.25)	1.05 (0.62)
SNIP_second_13–24y	**—**	**—**	**—**	**—**	0.00	10.61	0.00 (0.00‐0.70)	0.36 (0.69)
SNIP_second_25+y	**—**	**—**	**—**	**—**	0.00	8.43	1.00 (0.68‐1.28)	1.02 (0.74)
SNIP_senior_0–12y	**—**	**—**	**—**	**—**	0.00	10.26	0.81 (0.33‐1.09)	0.78 (0.67)
SNIP_senior_13–24y	**—**	**—**	**—**	**—**	0.00	1.59	0.00 (0.00‐0.00)	0.08 (0.21)
SNIP_senior_25+y	**—**	**—**	**—**	**—**	0.00	3.86	0.35 (0.00‐1.00)	0.56 (0.66)
Mentor level^e^	**—**	**—**	**—**	**—**	1.00	5.00	5.00 (5.00‐5.00)	4.71 (0.86)
Elite reliance^f^	**—**	**—**	**—**	**—**	1.00	10.00	1.37 (1.07‐1.67)	1.51 (0.68)
Selective mobilization capacity^g^	**—**	**—**	**—**	**—**	0.00	6.00	1.25 (1.00‐1.60)	1.30 (0.63)

a A currency exchange rate of JP ¥1=US $0.0067 is applicable.

b These variables were quantified based on The Times Higher Education World University Rankings 2023.

c Not applicable.

d SNIP: Source Normalized Impact per Paper.

e These variables were quantified based on the size of the Grant-in-Aid for Scientific Research projects secured by the mentor, with smaller values indicating greater competitiveness. The categories are coded as 1=Grant-in-Aid for Scientific Research on Innovative Areas, 2=the Grant-in-Aid for Specially Promoted Research, 3=Grant-in-Aid for Scientific Research (S), 4=Grant-in-Aid for Scientific Research (A), and 5=Grant-in-Aid for Scientific Research (B) or smaller.

f The ratio of the total number of principal investigator (PI)–participant collaborations to the number of distinct PIs across all projects in which the participant served as a project member. Larger values indicate higher level of reliance.

g The ratio of the total number of collaborator participations to the number of distinct collaborators across projects secured by the participant as a PI. The closer the value is to 1, the greater the team’s diversity; conversely, higher values indicate stronger ability to mobilize specific collaborators.

**Table 3.** Grant acquisition status as a principal investigator by phenotype clusters.

Metrics	All	Phenotype clusters					
		Group 1	Group 2	Group 3	Group 4	Group 5	Group 6
N_1_ (n [female], %)	1152 (87, 7.55)	11 (1, 9.1)	3 (0, 0.0)	26 (1, 3.8)	46 (3, 6.5)	295 (21, 7.12)	771 (61, 7.91)
Career length (y), median (IQR)	26 (21‐30)	32 (29‐31)	22 (17‐20)	33 (27‐30)	31 (22‐27)	32 (23‐27)	29 (21‐25)
Funding_0–12y (million US $^a^), median (IQR)	0.09 (0.03‐0.19)	0.00 (0.00‐0.00)	6.65 (5.32‐6.69)	0.00 (0.00‐0.31)	0.00 (0.00‐0.42)	0.25 (0.00‐0.47)	0.06 (0.03‐0.13)
Total funding (million US $^a^), median (IQR)	0.33 (0.16‐0.85)	11.62 (11.00‐14.10)	9.80 (8.74‐10.00)	7.45 (6.64‐8.28)	4.72 (3.83‐4.82)	1.00 (0.82‐1.26)	0.20 (0.13‐0.33)

a A currency exchange rate of JP ¥1=US $0.0067 is applicable.

The distribution of personal attributes and research fields by phenotype cluster is summarized in Table 4. No differences were observed between clusters in gender, but a significant difference was found in the distribution of university rank (Fisher exact test, *P*<.05). Notably, participants in groups 1, 2, and 3, characterized by high funding acquisition capabilities, appeared regardless of the university ranks (graduated); within group 1, a total of 4 participants had graduated from top-tier universities, 3 had graduated from midtier universities, and 4 had graduated from lower-tier universities (Table 4).

**Table 4.** Personal attributes and research fields by phenotype cluster.

Metrics	All (N_1_=1152), n (%)	Phenotype clusters, n (%)						*P* value (Fisher exact test^b^)
		Group 1 (n=11)^a^	Group 2 (n=3)^a^	Group 3 (n=26)^a^	Group 4 (n=46)^a^	Group 5 (n=295)^a^	Group 6 (n=771)^a^	
Gender								.96
Male	1065 (92.45)	10 (91.0)	3 (100.0)	25 (96.2)	43 (93.5)	274 (92.88)	710 (92.09)	
Female	87 (7.55)	1 (9.0)	0 (0)	1 (3.8)	3 (6.5)	21 (7.12)	61 (7.91)	
University rank (currently affiliated)^c^								<.001
High (≤100)	51 (4.43)	4 (36.4)	0 (0)	4 (15.4)	5 (10.9)	22 (7.46)	16 (2.08)	
Medium (101‐600)	256 (22.22)	5 (45.5)	2 (66.7)	10 (38.5)	17 (37.0)	80 (27.12)	142 (18.42)	
Low (≥600)	845 (73.35)	2 (18.2)	1 (33.3)	12 (46.2)	24 (52.2)	193 (65.42)	613 (79.51)	
University rank (graduated)^c^								<.001
High (≤100)	178 (15.45)	4 (36.4)	1 (33.3)	9 (34.6)	11 (23.9)	72 (24.41)	81 (10.51)	
Medium (101‐600)	383 (33.25)	3 (27.3)	1 (33.3)	11 (42.3)	19 (41.3)	120 (40.68)	229 (29.7)	
Low (≥600)	591 (51.3)	4 (36.4)	1 (33.3)	6 (23.1)	16 (34.8)	103 (34.92)	461 (59.79)	
Fields								<.001
Basic medicine	369 (32.03)	9 (81.8)	1 (33.3)	19 (73.1)	32 (69.6)	115 (38.98)	193 (25.03)	
Clinical medicine (internal medicine)	229 (19.88)	2 (18.2)	1 (33.3)	3 (11.5)	8 (17.4)	42 (14.24)	173 (22.44)	
Clinical medicine (surgery)	338 (29.34)	0 (0)	0 (0)	3 (11.5)	3 (6.5)	80 (27.12)	252 (32.68)	
Social medicine	62 (5.38)	0 (0)	1 (33.3)	0 (0)	1 (2.2)	15 (5.08)	45 (5.84)	
Nursing	59 (5.12)	0 (0)	0 (0)	0 (0)	0 (0)	9 (3.05)	50 (6.49)	
Dentistry	95 (8.25)	0 (0)	0 (0)	1 (3.8)	2 (4.3)	34 (11.53)	58 (7.52)	

a The characteristics and median total funding amounts for each group are as follows: group 1, established type, US $11.62 million; group 2, emerging type, US $9.80 million; group 3, catching-up trajectory type, US $7.45 million; group 4, plateaued type, US $4.72 million; group 5, moderate-level stable type, US $1.00 million; and group 6, low-slope stable type, US $0.20 million.

b Groups 1 and 2 were combined because of sparse cell counts. *P* values were calculated using Fisher exact test with Monte Carlo simulation (5000 simulations).

c These variables were quantified based on The Times Higher Education World University Rankings 2023.

Research fields were categorized into 6 areas: basic medicine, clinical medicine (internal medicine), clinical medicine (surgery), social medicine, nursing, and dentistry (Table 4). Basic medicine and clinical medicine (both internal medicine and surgery) accounted for the largest proportion of participants because the analysis focused on professors affiliated with medical schools and excluded researchers from independent nursing or dental schools. Public health studies are less developed in Japan than in some other countries and do not constitute an independent faculty member. The distribution of researchers securing high funding varied by field (Fisher exact test, *P*<.01). Group 1 participants were only observed in basic medicine and internal medicine (Table 4). In surgery and dentistry, group 3 received the highest total funding, whereas in social medicine, group 2 ranked highest (Table 4). None of the nursing researchers secured high levels of research funding, defined as group 4 or higher, with a median total grant amount of US $4.72 (IQR US $3.83‐US $4.82) million (JP ¥1 [US $0.0067]). These results suggest field-specific differences in the upper funding level ranges.

To examine differences in behavioral characteristics by field, we compiled indicator values (n=804; Table 5). Significant differences were found across fields for all indicators, revealing distinct research practices between fields (ANOVA, *P*<.05). Basic medicine and internal medicine were characterized by high-quality outcomes early in careers (SNIP_0–5y: basic medicine, mean 1.86, SE 0.11; and internal medicine: mean 1.86, SE 0.12) and throughout their careers (average SNIP: basic medicine: mean 0.94, SE 0.03; internal medicine: mean 0.89, SE 0.03), while basic medicine secured more large-scale research grants (# large-scale projects: mean 0.28, SE 0.05). Internal medicine, surgery, and social medicine demonstrated high productivity (total publication counts: internal medicine, mean 197.58, SE 11.11; surgery, mean 189.33, SE 8.88; social medicine, mean 199.40, SE 22.70). Social medicine was characterized by high initial productivity (# first-authored_0–5y: mean 5.19, SE 1.12), high collaborator diversity—indicated by low values for the following metrics (elite reliance: mean 1.34, SE 0.05; selective mobilization capacity: mean 1.15, SE 0.08)—and frequent implementation of large-scale projects (# large-scale projects: mean 0.25, SE 0.09). The use of complementary research programs was more common among nurses (% challenging research: mean 0.26, SE 0.08). In dentistry, the same researchers frequently collaborated on multiple projects (as indicated by high values for elite reliance: mean 1.72, SE 0.09 and selective mobilization capacity: mean 1.49, SE 0.09).

**Table 5.** Behavioral characteristics by fields (N_2_=804).

Metrics	Phenotype clusters, mean (SE)						ANOVA *P* value
	Basic medicine (n=240)	Clinical medicine (internal medicine; n=162)	Clinical medicine (surgery; n=247)	Social medicine (n=48)	Nursing (n=54)	Dentistry (n=53)	
Mentor level^a^	4.43 (0.07)	4.7 (0.07)	4.87 (0.04)	4.88 (0.09)	4.94 (0.03)	4.77 (0.1)	<.001
# first-authored_0–5y	3.6 (0.21)	3.96 (0.37)	3.67 (0.25)	5.19 (1.12)	1.3 (0.28)	2.45 (0.35)	<.001
SNIP_0–5y	1.86 (0.11)	1.86 (0.12)	1.35 (0.08)	1.41 (0.2)	0.44 (0.1)	1.22 (0.16)	<.001
Elite reliance	1.62 (0.06)	1.48 (0.04)	1.45 (0.03)	1.34 (0.05)	1.38 (0.05)	1.72 (0.09)	.002
Selective mobilization capacity	1.21 (0.04)	1.28 (0.06)	1.38 (0.03)	1.15 (0.08)	1.38 (0.07)	1.49 (0.09)	.004
Total publication counts	128.58 (6.34)	197.97 (11.11)	189.33 (8.88)	199.40 (22.70)	48.46 (9.19)	119.3 (10.48)	<.001
% lead-authored publications	0.56 (0.01)	0.52 (0.01)	0.53 (0.01)	0.51 (0.03)	0.69 (0.05)	0.55 (0.03)	<.001
Average SNIP^b^	0.94 (0.03)	0.89 (0.03)	0.75 (0.02)	0.79 (0.04)	0.62 (0.05)	0.82 (0.04)	<.001
# projects	8.20 (0.31)	6.49 (0.33)	5.68 (0.19)	5.25 (0.46)	5.07 (0.34)	7.32 (0.5)	<.001
# large-scale projects	0.28 (0.05)	0.12 (0.04)	0.09 (0.03)	0.25 (0.09)	0.11 (0.05)	0 (0)	.001
% challenging research	0.12 (0.02)	0.14 (0.02)	0.19 (0.02)	0.18 (0.04)	0.26 (0.07)	0.16 (0.04)	.04

a These variables were quantified based on the size of the Grant-in-Aid for Scientific Research projects secured by the mentor, with smaller values indicating greater competitiveness. The categories are coded as 1=Grant-in-Aid for Scientific Research on Innovative Areas, 2=the Grant-in-Aid for Specially Promoted Research, 3=Grant-in-Aid for Scientific Research (S), 4=Grant-in-Aid for Scientific Research (A), and 5=Grant-in-Aid for Scientific Research (B) or smaller.

b SNIP: Source Normalized Impact per Paper.

### Associations Between Behavioral Characteristics and Funding Acquisition Capabilities by Field

We examine the association between behavioral characteristics and funding acquisition using univariate logistic regression analyses conducted separately for each field (Table 6). Researchers in group 6 were used as the reference group, and all other types were pooled into a single comparison group (Table 6). Several common patterns emerged regarding the factors associated with substantial funding acquisition.

**Table 6.** Associations between behavioral characteristics and funding acquisition by fields (2-tailed *χ*^2^ test)^a^.

Variable and fields	Odds ratio (95% CI)	Significance of the model, *P* value (Wald test)
Mentor level (N_2_=804)^b^		
Basic medicine (n=240)	0.49 (0.36‐0.66)^c^	<.001^c^
Internal medicine (n=162)	0.67 (0.46‐0.96)^d^	.03^d^
Surgery (n=247)	0.38 (0.22‐0.68)^c^	.001^e^
Social medicine (n=48)	0.25 (0.02‐2.70)	.26
Nursing (n=54)	0.32 (0.03‐3.99)	.38
Dentistry (n=53)	0.63 (0.29‐1.39)	.25
# first-authored_0–5y (N_2_=804)		
Basic medicine (n=240)	1.06 (0.98‐1.15)	.14
Internal medicine (n=162)	1.02 (0.95‐1.10)	.58
Surgery (n=247)	1.00 (0.92‐1.08)	.98
Social medicine (n=48)	1.02 (0.94‐1.10)	.65
Nursing (n=54)	1.18 (0.86‐1.61)	.31
Dentistry (n=53)	0.96 (0.75‐1.24)	.78
SNIP_0–5y^f^ (N_2_=804)		
Basic medicine (n=240)	1.48 (1.22‐1.79)^c^	<.001^c^
Internal medicine (n=162)	1.34 (1.06‐1.69)^d^	.02^d^
Surgery (n=247)	1.70 (1.28‐2.27)^c^	.000^c^
Social medicine (n=48)	1.6 (0.98‐2.59)	.06
Nursing (n=54)	1.7 (0.65‐4.44)	.28
Dentistry (n=53)	1.58 (0.88‐2.83)	.13
Elite reliance (N_2_=804)		
Basic medicine (n=240)	0.99 (0.76‐1.28)	.92
Internal medicine (n=162)	1.20 (0.63‐2.29)	.58
Surgery (n=247)	2.09 (1.10‐3.97)^d^	.03^d^
Social medicine (n=48)	9.09 (1.32‐62.57)^d^	.03^d^
Nursing (n=54)	6.60 (1.02‐42.66)^d^	.047^d^
Dentistry (n=53)	1.01 (0.4‐2.54)	.99
Selective mobilization capacity (N_2_=804)		
Basic medicine (n=240)	1.15 (0.78‐1.71)	.48
Internal medicine (n=162)	1.44 (0.91‐2.28)	.12
Surgery (n=247)	1.84 (1.00‐3.38)	.05
Social medicine (n=48)	2.91 (0.71‐11.89)	.14
Nursing (n=54)	2.77 (0.58‐13.29)	.20
Dentistry (n=53)	5.98 (1.42‐25.21)^d^	.01^d^
Total publication count (N_2_=804)		
Basic medicine (n=240)	1.00 (1.00‐1.00)	.68
Internal medicine (n=162)	1.00 (1.00‐1.00)	.26
Surgery (n=247)	1.00 (0.998‐1.003)	.70
Social medicine (n=48)	1.01 (1.00‐1.01)^d^	.03^d^
Nursing (n=54)	1.01 (1.00‐1.02)^d^	.04^d^
Dentistry (n=53)	1.00 (1.00‐1.01)	.57
% lead-authored publications (N_2_=804)		
Basic medicine (n=240)	2.07 (0.64‐6.69)	.23
Internal medicine (n=162)	4.47 (0.61‐32.78)	.14
Surgery (n=247)	2.5 (0.58‐10.79)	.22
Social medicine (n=48)	3.11 (0.14‐67.89)	.47
Nursing (n=54)	1.53 (0.21‐11.28)	.67
Dentistry (n=53)	5.09 (0.17‐152.02)	.35
Average SNIP (N_2_=804)		
Basic medicine (n=240)	5.99 (2.63‐13.65)^c^	<.001^c^
Internal medicine (n=162)	3.17 (1.16‐8.70)^d^	.03^d^
Surgery (n=247)	9.79 (3.26‐29.43)^c^	<.001^c^
Social medicine (n=48)	1.00 (0.09‐11.84)	.998
Nursing (n=54)	0.71 (0.07‐6.98)	.77
Dentistry (n=53)	10.76 (1.09‐105.96)^d^	.04^d^
# large-scale projects (N_2_=804)		
Basic medicine (n=240)	25.41 (3.53‐183.03)^c^	.001^c^
Internal medicine (n=162)	—^g^	.99
Surgery (n=247)	24.27 (5.16‐114.09)^c^	<.001^c^
Social medicine (n=48)	9.67 (1. 98‐47.34)^c^	.005^e^
Nursing (n=54)	—^g^	.99
Dentistry (n=53)	—^g^	.99
% challenging research (N_2_=804)		
Basic medicine (n=240)	0.66 (0.26‐1.71)	.40
Internal medicine (n=162)	0.59 (0.10‐3.56)	.57
Surgery (n=247)	2.03 (0.90‐4.60)	.09
Social medicine (n=48)	12.67 (1.13‐142.10)^d^	.04^d^
Nursing (n=54)	0.68 (0.11‐4.25)	.68
Dentistry (n=53)	1.37 (0.23‐8.30)	.73
SNIP_first_0‐12y (N_2_=804)		
Basic medicine (n=240)	1.17 (0.88‐1.57)	.28
Internal medicine (n=162)	1.00 (0.62‐1.63)	.99
Surgery (n=247)	1.56 (1.04‐2.35)^d^	.03^d^
Social medicine (n=48)	0.97 (0.29‐3.27)	.97
Nursing (n=54)	1.12 (0.46‐2.72)	.81
Dentistry (n=53)	1.10 (0.61‐1.99)	.76
SNIP_first_13‐24y (N_2_=804)		
Basic medicine (n=240)	0.89 (0.45‐1.77)	.74
Internal medicine (n=162)	1.10 (0.3‐4.10)	.88
Surgery (n=247)	3.01 (0.89‐10.14)	.08
Social medicine (n=48)	4.82 (0.36‐64.39)	.23
Nursing (n=54)	6.87 (0.62‐76.69)	.12
Dentistry (n=53)	0.60 (0.13‐2.84)	.52
SNIP_first_25+y (N_2_=804)		
Basic medicine (n=240)	1.11 (0.98‐1.25)	.10
Internal medicine (n=162)	1.10 (0.92‐1.31)	.31
Surgery (n=247)	0.96 (0.76‐1.20)	.70
Social medicine (n=48)	0.92 (0.61‐1.39)	.68
Nursing (n=54)	0.85 (0.22‐3.33)	.81
Dentistry (n=53)	1.00 (0.71‐1.41)	.99
SNIP_second_0‐12 y (N_2_=804)		
Basic medicine (n=240)	1.79 (1.16‐2.75)	.008
Internal medicine (n=162)	3.31 (1.6‐6.86)^c^	.001^c^
Surgery (n=247)	2.22 (1.18‐4.17)^d^	.01^d^
Social medicine (n=48)	2.50 (0.56‐11.11)	.23
Nursing (n=54)	1.55 (0.38‐6.30)	.54
Dentistry (n=53)	2.2 (0.68‐7.16)	.19
SNIP_second_13‐24 y (N_2_=804)		
Basic medicine (n=240)	1.36 (0.85‐2.17)	.21
Internal medicine (n=162)	0.77 (0.44‐1.33)	.35
Surgery (n=247)	1.11 (0.67‐1.81)	.69
Social medicine (n=48)	1.61 (0.52‐5.03)	.41
Nursing (n=54)	1.45 (0.27‐7.70)	.67
Dentistry (n=53)	1.42 (0.40‐5.05)	.59
SNIP_second_25+ y (N_2_=804)		
Basic medicine (n=240)	2.43 (1.59‐3.70)^c^	<.001^c^
Internal medicine (n=162)	1.09 (0.75‐1.58)	.66
Surgery (n=247)	2.17 (1.16‐4.04)^d^	.02^d^
Social medicine (n=48)	1.60 (0.44‐5.80)	.47
Nursing (n=54)	1.60 (0.42‐6.01)	.49
Dentistry (n=53)	4.80 (1.07‐21.55)^d^	.04^d^
SNIP_senior_0‐12y (N_2_=804)		
Basic medicine (n=240)	3.12 (1.89‐5.16)^c^	<.001^c^
Internal medicine (n=162)	2.61 (1.43‐4.76)^e^	.002^e^
Surgery (n=247)	2.03 (1.15‐3.58)^d^	.02^d^
Social medicine (n=48)	11.66 (1.75‐77.73)^d^	.01^d^
Nursing (n=54)	1.96 (0.55‐6.95)	.30
Dentistry (n=53)	1.75 (0.58‐5.29)	.32
SNIP_senior_13‐24y (N_2_=804)		
Basic medicine (n=240)	2.49 (0.71‐8.81)	.16
Internal medicine (n=162)	1.20 (0.27‐5.39)	.81
Surgery (n=247)	1.39 (0.35‐5.55)	.64
Social medicine (n=48)	7.28 (0.35‐151.28.20)	.20
Nursing (n=54)	2.12 (0.01‐314.94)	.77
Dentistry (n=53)	1.40 (0.14‐13.59)	.77
SNIP_senior_25+y (N_2_=804)		
Basic medicine (n=240)	2.15 (1.46‐3.17)^c^	<.001^c^
Internal medicine (n=162)	2.09 (1.27‐3.45)^e^	.004^e^
Surgery (n=247)	1.77 (1.06‐2.96)^d^	.03^d^
Social medicine (n=48)	1.15 (0.39‐3.39)	.80
Nursing (n=54)	0.74 (0.11‐5.18)	.76
Dentistry (n=53)	0.82 (0.29‐2.3)	.70

a The researcher type was used as the dependent variable. Group 6 was used as a reference.

b These variables were quantified based on the largest grant category secured by the mentor, with smaller values indicating greater competitiveness.

c <.001.

d <.05.

e <.01.

f SNIP: Source Normalized Impact per Paper.

g No results were produced by XLSTAT software for this group.

For the majority of participants (basic medicine, internal medicine, and surgery), becoming a high-funding recipient required early success. Specifically, higher SNIP_0–5y values were positively associated with becoming a high-funding recipient in basic medicine (odds ratio [OR] 1.48, 95% CI 1.22‐1.79), internal medicine (OR 1.34, 95% CI 1.06‐1.69), and surgery (OR 1.70, 95% CI 1.28‐2.27). Likewise, researchers whose mentors had higher competitiveness scores were more likely to become high-funding recipients, as indicated by the inverse association with the mentor-level score (basic medicine: OR 0.49, 95% CI 0.36‐0.66; internal medicine: OR 0.67, 95% CI 0.46‐0.96; surgery: OR 0.38, 95% CI 0.22‐0.68). Sustained high-quality leadership outputs were also significant (average SNIP: basic medicine, OR 5.99, 95% CI 2.63‐13.65; internal medicine, OR 3.17, 95% CI 1.16‐8.70; surgery, OR 9.79, 95% CI 3.26‐29.43; Table 6). This was further supported by the results of our analysis that explored factors characterizing high-funding recipients without distinguishing fields (Multimedia Appendices 3 and 4).

For surgery, in addition to these factors, supporting specific collaborators (surgery: elite reliance: OR 2.09, 95% CI 1.10‐3.97) and the quality of publications during the first 12 years of a researcher’s career were major determinants of success (surgery: SNIP_first_0–12y, OR 1.56, 95% CI 1.04‐2.35; SNIP_second_0–12y: OR 2.22, 95% CI 1.18‐4.17; Table 6).

As funding acquisition data were characterized by small amounts for most researchers and large outliers for a limited number of researchers, some estimates showed wide CIs. Therefore, these associations should be regarded as exploratory. Nevertheless, positive associations (Multimedia Appendix 5) with high funding were observed for elite reliance in the field of social medicine (OR 9.09, 95% CI 1.32‐62.57), early-career senior-author impact (SNIP_senior_0‐12y: OR 11.66, 95% CI 1.75‐77.73), and engagement in challenging research (% challenging research: OR 12.67, 95% CI 1.13‐142.10; Table 6). In dentistry, selective mobilization capacity (OR 5.98, 95% CI 1.42‐25.21) and late-career second-author impact (SNIP_second_25+y: OR 4.80, 95% CI 1.07‐21.55) were also positively associated with high funding (Table 6).

To assess the robustness of funding acquisition patterns, we conducted multiple linear regressions using personal attributes and behavioral characteristics. As total funding raised (indicator: total funding) was inherently related to the total number of funded projects (indicator: # projects), the latter variable was excluded from the regression analyses to avoid structural redundancy. Consistent with this relationship, the 2 variables were strongly correlated (*r*=0.847; Multimedia Appendix 6).

To mitigate concerns regarding reverse causality and the influence of unmeasured variables, we first constructed models using theoretically relevant covariates for each career stage and assessed multicollinearity prior to model selection (Table 7). Model 1 included variables related to personal attributes and early-career activities. In model 2, we additionally included early-career funding acquisition to account for the cumulative effects of prior funding success. Following the inclusion of this variable, the coefficient for graduate university rank changed direction but remained close to zero and statistically nonsignificant (from −0.02±0.04 to 0.02±0.03; not significant; Table 7), suggesting minimal independent association with subsequent funding acquisition. As the influence of graduate university rank appeared to be limited and largely accounted for by early-career funding success, this variable was not retained in subsequent models.

**Table 7.** Comparison of sequential regression model specifications for funding acquisition.^a^

Metrics	Model 1 (N_2_=804)	Model 2 (N_2_=804)	Model 3 (N_2_=804)	Model 4 (N_2_=804)
Career length, standardized coefficient (SE)	0.11 (0.03)^b^	0.14 (0.03)^b^	0.15 (0.03)^b^	0.09 (0.03)^c^
University rank (currently affiliated), standardized coefficient (SE)^d^	−0.19 (0.04)^b^	−0.19 (0.03)^b^	−0.18 (0.03)^b^	−0.16 (0.03)^b^
University rank (graduated), standardized coefficient (SE)^d^	−0.02 (0.04)	0.02 (0.03)	—^e^	—
Mentor level, standardized coefficient (SE)^f^	−0.27 (0.03)^b^	−0.24 (0.03)^b^	0.12 (0.03)^b^	0.12 (0.04)^b^
# first-authored_0–5y, standardized coefficient (SE)	−0.02 (0.03)	−0.02 (0.03)	−0.21 (0.03)^b^	−0.21 (0.03)^b^
SNIP_0–5y, standardized coefficient (SE)	0.21 (0.04)^b^	0.17 (0.03)^b^	−0.03 (0.03)	−0.04 (0.03)
Funding_0–12y, standardized coefficient (SE)	—	0.32 (0.03)^b^	0.32 (0.03)^b^	0.32 (0.03)^b^
% lead-authored publications, standardized coefficient (SE)	—	—	0.14 (0.03)^b^	0.07 (0.03)^c^
Average SNIP, standardized coefficient (SE)^g^	—	—	0.09 (0.03)^h^	—
SNIP_second_0‐12y, standardized coefficient (SE)	—	—	—	0.03 (0.03)
SNIP_second_13‐24y, standardized coefficient (SE)	—	—	—	−0.02 (0.03)
SNIP_second_25+y, standardized coefficient (SE)	—	—	—	0.04 (0.03)
SNIP_senior_0‐12y, standardized coefficient (SE)	—	—	—	0.12 (0.03)^b^
SNIP_senior_13‐24y, standardized coefficient (SE)	—	—	—	0.1 (0.03)^h^
SNIP_senior_25+y, standardized coefficient (SE)	—	—	—	0.06 (0.03)
Male, standardized coefficient (SE)	−0.02 (0.03)	−0.03 (0.03)	−0.03 (0.03)	−0.03 (0.03)
Female, standardized coefficient (SE)	0 (0)	0 (0)	0 (0)	0 (0)
VIF^i^	1.046	1.103	1.120	1.132
*F* test (*df*)	30.364 (7, 796)	43.806 (8, 795)	42.878 (9, 794)	29.255 (14, 789)
Adjusted *R*²	0.211	0.299	0.319	0.330
AIC^j^	16115.889	16014.542	15991.702	15984.003

a The dependent variable was the sum of the amount allocated for each project obtained as principal investigator.

b *P*<.001.

c *P*<.05.

d These variables were quantified based on The Times Higher Education World University Rankings 2023, with smaller values indicating higher ranking.

e Not applicable.

f These variables were quantified based on the largest grant category secured by the mentor, with smaller values indicating greater competitiveness.

g SNIP: Source Normalized Impact per Paper.

h *P*<.01.

i VIF: variance inflation factor.

j AIC: Akaike information criterion.

Model 3 further incorporated variables reflecting research activity across the entire career (indicators: % lead-authored publications and average SNIP). Following the inclusion of these variables, the coefficient for mentor level reversed direction while remaining statistically significant—it changed from −0.24±0.03 to 0.12±0.03 (*P*<.001). The coefficient for early success became significant for the number of papers (indicator: # first-authored_0–5y, −0.02±0.03 to −0.21±0.03; *P*<.001), whereas the coefficient for impact lost statistical significance (indicator: SNIP_0–5y: 0.17±0.03 to −0.03±0.03; *P*>.05). This suggests that part of the influence of early-career variables may be mediated through subsequent career experiences. To reflect the temporal structure of career development and clarify how associations change across career stages, we decided to retain the early-career variables in the model. In model 3, variables related to collaborative research relationships and acquisition strategies were excluded (indicators: elite reliance, selective mobilization capacity, # large-scale projects, and % challenging research; Table 7) because their univariate associations demonstrated wide CIs and appeared to be strongly influenced by extreme outliers (Table 6).

Finally, model 4 replaced the career-wide research quality variable used in model 3 (indicator: average SNIP) with research quality variables stratified by career stage and author position within 12-year career intervals (Table 7). As model 1 already captured early-career activity patterns as a first-author during the first 5 years (indicator: # first-authored_0–5y and SNIP_0–5y), additional variables representing first-author activity across the first 12 career years were not included in model 4 to avoid redundancy among temporally overlapping indicators. Furthermore, first-author activity indicators from the midcareer stage onward were excluded because their univariate associations were not statistically significant (Table 6). Therefore, only variables representing second-author and senior-author activity from the midcareer stage onward were added.

After confirming acceptable variance inflation factor values across alternative model specifications (Table 7), model 4 was selected as the final modeling framework because it allowed stage-specific research activity indicators to be evaluated while minimizing redundancy among temporally overlapping variables. Field-specific regression models were subsequently constructed based on this framework (Table 8). The model fit, evaluated with adjusted *R*^2^ values, was high for social medicine and dentistry with low Akaike information criterion (AIC) values, suggesting that funding acquisition in these fields can be explained and predicted with considerable accuracy (model 4: adjusted *R*^2^=0.890; model 6: adjusted *R*^2^=0.738; Table 8). In surgery, an adjusted *R*² of 0.576 indicated a reasonably good predictive performance, although a high AIC suggested limited justification for retaining variables with nonsignificant coefficients. Conversely, the results for nurses showed limited explanatory power, suggesting that key determinants in this field might not have been fully captured (model 5: *F* test not significant; adjusted *R*²=0.047; Table 8). For basic medicine and internal medicine, relatively low adjusted *R*² values and high AIC values suggested that additional factors outside this analysis may influence funding acquisition (model 1: adjusted *R*^2^=0.288; model 2: adjusted *R*^2^=0.318; Table 8).

**Table 8.** Probability of funding acquisition by field (based on a 2-tailed *t* test; ordinary least squares was used for analysis)^a^.

Metrics	Field					
	Basic medicine (n=240)	Clinical medicine (internal medicine; n=162)	Clinical medicine (surgery; n=247)	Social medicine (n=48)	Nursing (n=54)	Dentistry (n=53)
Career length, standardized coefficient (SE)	0.10 (0.07)	0.09 (0.08)	0.16 (0.05)^b^	0.20 (0.08)^c^	0.22 (0.19)	0.02 (0.11)
University rank (currently affiliated), standardized coefficient (SE)^d^	0.21 (0.06)^e^	0.38 (0.07)^e^	0.52 (0.04)^e^	0.91 (0.07)^e^	0.03 (0.16)	0.39 (0.09)^e^
Mentor level, standardized coefficient (SE)^f^	−0.25 (0.06)^e^	−0.24 (0.07)^e^	−0.07(0.04)	−0.07 (0.06)	−0.49 (0.2)	−0.03 (0.08)
# first-authored_0–5y, standardized coefficient (SE)	−0.19 (0.06)^b^	−0.15 (0.07)^c^	−0.34 (0.05)^e^	0.01 (0.06)	−0.20 (0.15)	0.04 (0.09)
SNIP_0–5y, standardized coefficient (SE)	−0.06 (0.06)	0.02 (0.08)	−0.07 (0.05)	0.00 (0.06)	0.16 (0.24)	−0.22 (0.09)^c^
Funding_0–12y, standardized coefficient (SE)	0.11 (0.07)	−0.05 (0.08)	0.20 (0.05)^e^	−0.01 (0.08)	−0.12 (0.28)	0.46 (0.11)^e^
% lead-authored publications, standardized coefficient (SE)	0.08 (0.06)	0.11 (0.07)	0.01 (0.05)	0.06 (0.06)	−0.02 (0.15)	0.04 (0.09)
SNIP_second_0‐12y, standardized coefficient (SE)	0.02 (0.06)	0.05 (0.07)	0.03 (0.05)	0.15 (0.07)^c^	−0.02 (0.18)	0.39 (0.1)^e^
SNIP_second_13‐24y, standardized coefficient (SE)	0.03 (0.06)	−0.05 (0.07)	−0.04 (0.05)	−0.10 (0.07)	−0.21 (0.19)	0.24 (0.1)^c^
SNIP_second_25+y, standardized coefficient (SE)	0.03 (0.06)	0.07 (0.07)	0.07 (0.05)	0.04 (0.07)	−0.05 (0.17)	0.17 (0.09)
SNIP_senior_0‐12y, standardized coefficient (SE)	0.20 (0.06)^e^	0.11 (0.07)	0.02 (0.05)	−0.04 (0.07)	0.17 (0.17)	0.05 (0.09)
SNIP_senior_13‐24y, standardized coefficient (SE)	0.04 (0.06)	0.24 (0.08)^b^	0.06 (0.05)	0.20 (0.06)^b^	−0.16 (0.16)	−0.02 (0.1)
SNIP_senior_25+y, standardized coefficient (SE)	0.04 (0.06)	0.10 (0.07)	0.04 (0.05)	−0.01 (0.06)	−0.08 (0.15)	−0.14 (0.08)
Male, standardized coefficient (SE)	−0.06 (0.06)	0.05 (0.07)	−0.01 (0.04)	−0.03 (0.05)	−0.11 (0.18)	0.03 (0.09)
Female, standardized coefficient (SE)	0 (0)	0 (0)	0 (0)	0 (0)	0 (0)	0 (0)
*F* test (*df*)	7.902 (14, 225)^e^	6.368 (14, 147)^e^	24.910 (14, 232)^e^	28.280 (14, 33)^e^	0.322 (14, 39)	11.441 (15, 38)^e^
Adjusted *R*²	0.288	0.318	0.576	0.890	0.047	0.738
AIC^g^	4976.318	3217.847	4371.011	876.836	932.875	919.093

a The dependent variable was the sum of the amount allocated for each project obtained as principal investigator.

b *P*<.01.

c *P*<.05.

d These variables were quantified based on The Times Higher Education World University Rankings 2023, with smaller values indicating higher ranking.

e *P*<.001.

f These variables were quantified based on the largest grant category secured by the mentor, with smaller values indicating greater competitiveness.

g AIC: Akaike information criterion.

## Discussion

### Principal Findings

Funding disparities across academic fields have been analyzed through publication-linked funding information, showing both the breadth and concentration of funding distribution within medical subfields [25,26]. However, because these are research evaluations rather than assessments of individual achievements, they do not sufficiently analyze the differences in researchers’ abilities to secure funding. The analysis demonstrated that the maximum funding secured by individual researchers varies by field.

One possible explanation for the limited number of high-funding recipients in nursing is the study design itself. While the initial data extraction yielded a sample believed to reflect the structure of the population, subsequent procedures—specifically the inclusion criteria of being affiliated with a national university medical school and having obtained a doctoral degree since 1996—excluded researchers from private universities with well-established nursing programs, as well as many experienced nursing researchers whose scholarship is grounded primarily in extensive clinical practice (from 9.96% to 5.12%; Table 1). On the other hand, observations from researchers affiliated with Japanese medical schools suggest that highly funded nursing researchers are often leaders who have created and are driving emerging subfields such as patient-participatory nursing and nursing digital transformation. Consequently, the research population may be smaller in these new subfields. This may partly explain the limited number of recipients of very large-scale research funding in nursing.

The scarcity of high-funding recipients in surgery and dentistry (Table 4) may reflect the historical origins of these practice-oriented professions and their field cultures that traditionally value technical crafts. Clinically oriented publications receive fewer citations than those focused on basic science, making funding acquisition more difficult [27]. While low citation counts may reflect a narrow readership due to high specialization, there is evidence that lower-prestige publications may hinder the diffusion of cross-disciplinary ideas [28]. US NIH data indicate that selecting themes favored by a broader scientific community (ie, more fundamental or mechanistic themes) can influence funding decisions regardless of the quality of future outcomes [29]. In Japan’s GIA large-scale projects, the broader range of reviewers’ specialties compared with small-scale projects may be one reason fewer high-funding recipients are found in surgery and dentistry. Greater clinical demands, driven by the growing importance of revenue-generating activities for university hospitals following the 2004 partial privatization reform of national universities, may also contribute [30].

This study showed the impact of individual efforts, such as demonstrating leadership to produce high-quality outcomes at each career stage (Table 6). The importance of second authorship, previously underexamined, indicates the degree of centralization of the research ecosystem in each field [31]. The finding that early success in surgery accounted for nearly the entire outcome (Table 8) suggests a strong influence of institutional and academic structures. However, the reversal of the coefficients for mentor level and early success after controlling for career-long achievements suggests that the apparent effects of these early-career factors may be partly attributable to their association with subsequent research performance (Table 7). Funding success therefore appears to reflect not only early-career advantages but also achievements accumulated throughout a research career.

Promoting complementary research while supporting specific collaborators in social medicine or maintaining a subleadership position while mobilizing specific researchers as supporters in dentistry (Table 6) can be regarded as evidence that researchers are taking proactive measures in these fields. The field-specific research design of this study, by excluding typical leaders who tend to attract an excessive concentration of funding in these fields, may have highlighted the importance of the actions of individuals with relatively less influence. Examining the moderate funding recipients and diverse research behaviors may be more informative than focusing solely on top recipients.

This study provides new insights into individual agency within the process through which researchers transition from peripheral to central roles. As effective individual actions and timing for securing funding vary by field, it is necessary to concentrate efforts at the most advantageous time (Table 6).

### Limitations

The limitations of this study are as follows. First, it does not cover all professors conducting research with GIA grants, as the analysis is restricted to researchers who obtained their doctoral degrees after 1996 and whose publication records can be traced from the early stages of their careers. Consequently, researchers in the oldest age group and those without doctoral degrees are excluded. However, this restriction is consistent with the purpose of the study. As Japan’s research funding system shifted toward a competitive funding model in 2004, focusing on researchers whose careers developed under this system provides an appropriate basis for examining its effects. Accordingly, the findings should be interpreted as reflecting trends within the post-2004, globally standardized research environment. The experiences of older researchers and those without doctoral degrees remain important topics for future research. In particular, historical comparisons could provide valuable insights into how research was nurtured within Japan’s pre-2004 research ecosystem.

Second, SciVal is a commercial database with limited journal coverage, and reliance on this source alone may underestimate researchers’ publication output. The extent of this underestimation may vary across fields. On the basis of our previous analysis of researcher career outcomes, SciVal data appear to reasonably capture researcher performance in fields with globally shared research interests, such as basic and clinical medicine [32]. In contrast, the limitations are more pronounced in fields where findings are frequently disseminated in local languages. Further discipline-specific investigations are needed. Furthermore, this analysis included all forms of research output to capture diverse scholarly contributions, including conceptual synthesis in review articles. While our preliminary analyses suggest that review articles are not strongly associated with grant acquisition, recent advances in AI and laboratory automation suggest the need to reevaluate scholarly contributions beyond traditional academic publications.

The third limitation concerns the indicators used in this study. While this study demonstrated that second authorship can serve as a stepping stone to a core role, the path to senior author excellence remains unclear. Future research should develop new metrics that capture the diverse contributions of middle authors to understand the determinants of core and peripheral roles [33,34]. Moreover, it could not account for aspirations to lead large-scale research projects, which may be partially reflected in their rates of submitting large-scale grant applications. Future analyses should incorporate variations in application behavior to better capture such ambitions. In addition, funding acquisition may also be influenced by finer behavioral differences, such as promotional language in proposals. Addressing these factors will require new behavioral measures beyond existing research evaluation metrics.

Finally, potential confounding between early-career variables and total-career indices should be considered when interpreting the results. Early-career variables were retained in the models because the attenuation of their effects after adjustment for total-career factors was itself a finding of interest. As a result, some confounding may remain, and the estimated coefficients should be interpreted with caution. Furthermore, residual confounding due to unmeasured factors cannot be excluded, limiting causal interpretation of the observed associations.

### Conclusions

This study identifies individual research-related behaviors that influence GIA funding acquisition across research fields. The results highlight that while organizational and academic structures have a significant impact, personal initiative during researchers’ transition from peripheral to core roles becomes important, emphasizing strong relationship-building skills. In other words, both participants and coordinators in collaborative research, commitment to reshaping research agendas, and excellent publication records as the lead author are crucial factors that affect funding acquisition. Effective actions and timing vary by field, reflecting the maturity of each field’s ecosystem. These survey results indicate the actions and timing that enable individual researchers to maximize funding acquisition and expand their research, providing a foundation for more effective institutional support strategies.

## Supplementary material

10.2196/98428 Multimedia Appendix 1 The metrics.

10.2196/98428 Multimedia Appendix 2 Phenotype clusters.

10.2196/98428 Multimedia Appendix 3 Behavioral characteristics by phenotype clusters.

10.2196/98428 Multimedia Appendix 4 Associations between behavioral characteristics and funding acquisition by phenotype clusters.

10.2196/98428 Multimedia Appendix 5 Regression of behavior-related indicators against the amount allocated (log-transformed).

10.2196/98428 Multimedia Appendix 6 Correlation matrix.

## Abbreviations

AIC: Akaike information criterion
GIA: Grant-in-Aid for Scientific Research
NIH: National Institutes of Health
OR: odds ratio
PI: principal investigator
SNIP: Source Normalized Impact per Paper
