# Supplementary material for: Individual Research Behaviors and Research Funding Acquisition Across Fields and Career Periods: Regression Analysis
Source: Interact J Med Res. 2026 Jul 27;15:e98428. doi: 10.2196/98428 (PMC13405367; doi:10.2196/98428)
Supplement: Multimedia Appendix 1 [file ijmr-v15-e98428-s001.pdf]

Multimedia Appendix 1. The metrics.

Metrics	Definition
Total funding	Sum of the amount allocated for each project.
Funding_0–12y	Funding as PI during the first 12 years after doctorate completion.
Career length	Number of years after doctorate completion
University rank (graduated)	Rank of the doctoral degree–granting institution. These variables were quantified based on The Times Higher Education World University Rankings 2023 and is classified into seven levels, with smaller numerical values indicating higher rankings: 1 = top 100, 2 = 201–600, 3 = 601–1000, 4 = 1001–1200, 5 = 1201–1500, 6 = ≥1501, and 7 = out of ranking.
University rank (currently affiliated)	Rank of the current affiliated institution. This variable is coded using the same scheme as University rank (graduated).
Mentor level	The largest grant category secured by the mentor under whom the participant first became a project member. The categories are coded as 1 = *Grant-in-Aid for Scientific Research on Innovative Areas*, 2 = the *Grant-in-Aid for Specially Promoted Research*, 3 = *Grant-in-Aid for Scientific Research (S)*, 4 = *Grant-in-Aid for Scientific Research (A)*, and 5 = *Grant-in-Aid for Scientific Research (B)* or smaller.
# first-authored_0–5y	Number of first-authored publications (0–5 years post-PhD).
SNIP_0–5y	Total SNIP of first-authored publications (0–5 years post-PhD).
# projects	Total number of projects obtained as PI.
# large-scale projects	Number of large-scale projects obtained as PI, including Grants-in-Aid for Specially Promoted Research and Scientific Research (S) and (A), classified as large-scale categories.
% challenging research	Percentage of challenging exploratory research projects obtained as PI, including the *Grant-in-Aid for Exploratory Research*, *Grant-in-Aid for Challenging Exploratory Research*, and *Grant-in-Aid for Challenging Research (Pioneering)* or *(Exploratory)*, which support explanatory research.
Elite reliance	Ratio of the cumulative to unique number of PIs across projects involving the participant as a project member.
Selective mobilization capacity	Ratio of the cumulative to unique number of collaborators across projects secured by the participants as a PI.
Total publication counts	Total publication counts
% lead-authored publications	Percentage of first-, second-, and senior-authored publications
Average SNIP	Average SNIP of first-, second-, and senior-authored publications
SNIP_first_0–12 y	Average SNIP of first-authored publications (0–12 years post-PhD)
SNIP_first_13-24 y	Average SNIP of first-authored publications (13–24 years post-PhD)
SNIP_first_25+ y	Average SNIP of first-authored publications (25+ years post-PhD)
SNIP_second_0–12 y	Average SNIP of second-authored publications (0–12 years post-PhD)
SNIP_second_13-24 y	Average SNIP of second-authored publications (13–24 years post-PhD)
SNIP_second_25+ y	Average SNIP of second-authored publications (25+ years post-PhD)

SNIP_senior_0–12 y	Average SNIP of senior-authored publications (0–12 years post-PhD)
SNIP_senior_13-24 y	Average SNIP of senior-authored publications (13–24 years post-PhD)
SNIP_senior_25+ y	Average SNIP of senior-authored publications (25+ years post-PhD)
