# Supplementary material for: Individual Research Behaviors and Research Funding Acquisition Across Fields and Career Periods: Regression Analysis
Source: Interact J Med Res. 2026 Jul 27;15:e98428. doi: 10.2196/98428 (PMC13405367; doi:10.2196/98428)
Supplement: Multimedia Appendix 2 [file ijmr-v15-e98428-s002.pdf]

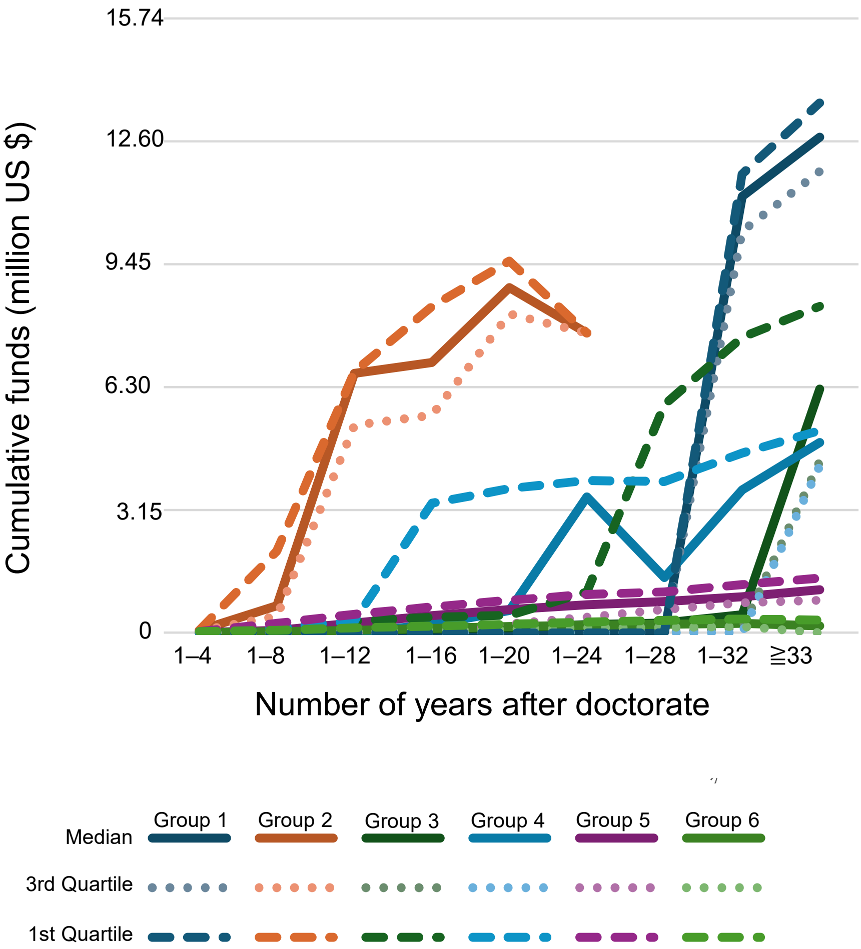

Multimedia Appendix 2. Funding acquisition patterns by phenotype cluster. Amount of GIA earned as PI within a specified period after doctorate completion was calculated and presented as a trend over time. Due to differences in career length, the number of the participants at each point varied. A currency exchange rate of JP ¥1=US $0.0067 is applicable
