# Supplementary material for: Individual Research Behaviors and Research Funding Acquisition Across Fields and Career Periods: Regression Analysis
Source: Interact J Med Res. 2026 Jul 27;15:e98428. doi: 10.2196/98428 (PMC13405367; doi:10.2196/98428)
Supplement: Multimedia Appendix 3 [file ijmr-v15-e98428-s003.pdf]

Multimedia Appendix 3. Behavioral characteristics by phenotype clusters.

	Phenotype clusters, mean (SE)						ANOVA *p*-value
	Group 1	Group 2	Group 3	Group 4	Group5	Group 6	
N = 804	n = 5	n = 3	n = 13	n = 29	n = 182	n = 572	
Mentor level [a]	2.4(0.51)	4.67(0.33)	3.77(0.47)	3.93(0.28)	4.43(0.08)	4.87(0.02)	<0.0001
# first-authored_0–5y	3.4(0.6)	9.67(2.33)	3.23(0.82)	3.66(0.64)	3.89(0.26)	3.42(0.18)	— [b]
SNIP_0–5y	3.59(1.32)	3.91(1.57)	2.69(0.71)	2.13(0.38)	2.13(0.15)	1.27(0.05)	<0.0001
Elite reliance	1.51(0.21)	1.06(0.06)	1.62(0.17)	1.44(0.12)	1.63(0.07)	1.48(0.02)	—
Selective mobilization capacity	1.18(0.07)	1.47(0.39)	1.16(0.17)	1.33(0.1)	1.42(0.05)	1.26(0.03)	—
Total publication counts	153.6(38.77)	146(67.09)	131.38(21.7)	169.48(24.07)	165.05(10.56)	157.93(5.32)	—
% lead-authored publications	0.66(0.09)	0.64(0.1)	0.67(0.07)	0.54(0.03)	0.57(0.02)	0.54(0.01)	—
Average SNIP	1.1(0.2)	1.06(0.04)	1.17(0.16)	1.16(0.08)	0.95(0.03)	0.78(0.01)	<0.0001
# projects	23.4(4.73)	11(1.53)	15.31(1.98)	12(1)	9.93(0.24)	4.95(0.09)	<0.0001
# large-scale projects	2.2(0.66)	0(0)	1.62(0.42)	0.72(0.19)	0.37(0.06)	0.01(0)	<0.0001
% challenging research	0.09(0.03)	0.07(0.04)	0.16(0.06)	0.18(0.04)	0.17(0.02)	0.16(0.01)	—

[a] These variables were quantified based on the size of the GIA projects secured by the mentor, with smaller values indicating greater competitiveness.

[b] *P*-values greater than 0.05 are represented by em dashes.
