# Supplementary material for: Individual Research Behaviors and Research Funding Acquisition Across Fields and Career Periods: Regression Analysis
Source: Interact J Med Res. 2026 Jul 27;15:e98428. doi: 10.2196/98428 (PMC13405367; doi:10.2196/98428)
Supplement: Multimedia Appendix 4 [file ijmr-v15-e98428-s004.pdf]

Multimedia Appendix 4. Associations between behavioral characteristics and funding acquisition by phenotype clusters (2-tailed χ2 test). [a]

Variable and clusters		Odds ratio (95% CI)	Significance of the model, *p* value (Wald test)
Mentor level (N=804) [b]			<0.0001 [c]
	Group 1 (n = 11)	0.23 [c] (0.13–0.40)	
	Group 2 (n = 3)	0.65(0.18–2.34)	
	Group 3 (n = 13)	0.37 [c] (0.25–0.54)	
	Group 4 (n = 29)	0.39 [c] (0.29–0.53)	
	Group 5 (n = 182)	0.52 [c] (0.42–0.64)	
# first-authored_0–5y (N=804)			<0.0001 [c]
	Group 1 (n = 11)	1.00(0.76–1.32)	
	Group 2 (n = 3)	1.44 [c] (1.27–1.63)	
	Group 3 (n = 13)	0.99(0.83–1.18)	
	Group 4 (n = 29)	1.01(0.91–1.13)	
	Group 5 (n = 182)	1.03(0.98–1.08)	
SNIP_0–5y (N=804)			<0.0001 [c]
	Group 1 (n = 11)	1.93 [c] (1.39–2.69)	
	Group 2 (n = 3)	2.01 [c] (1.35–2.98)	
	Group 3 (n = 13)	1.69 [c] (1.31–2.19)	
	Group 4 (n = 29)	1.49 [c] (1.21–1.85)	
	Group 5 (n = 182)	1.49 [c] (1.32–1.68)	
Elite reliance (N=804)			— [d]
	Group 1 (n = 11)	1.08(0.27–4.29)	
	Group 2 (n = 3)	0.00(0.00–19.24)	
	Group 3 (n = 13)	1.33(0.71–2.50)	
	Group 4 (n = 29)	0.89(0.44–1.81)	
	Group 5 (n = 182)	1.34 (1.06–1.68)	
Selective mobilization capacity (N=804)			—
	Group 1 (n = 11)	0.80(0.19–3.33)	
	Group 2 (n = 3)	1.64(0.33–8.17)	
	Group 3 (n = 13)	0.76(0.31–1.85)	
	Group 4 (n = 29)	1.18(0.65–2.13)	
	Group 5 (n = 182)	1.48 (1.14–1.93)	
Total publication count			—

Group 1 (n = 11)	1.00(0.99–1.01)	
Group 2 (n = 3)	1.00(0.99–1.01)	
Group 3 (n = 13)	1.00(1.00–1.00)	
Group 4 (n = 29)	1.00(1.00–1.00)	
Group 5 (n = 182)	1.00(0.99–1.00)	
% lead-authored publications (N=804)		—
Group 1 (n = 11)	7.83(0.31–195.51)	
Group 2 (n = 3)	5.85(0.08–438.45)	
Group 3 (n = 13)	9.65(1.34–69.68)	
Group 4 (n = 29)	1.03(0.19–5.69)	
Group 5 (n = 182)	2.07 (0.99–4.33)	
Average SNIP (N=804)		<0.0001 [c]
Group 1 (n = 11)	10.22 [e] (1.75–59.75)	
Group 2 (n = 3)	8.61(0.78–94.63)	
Group 3 (n = 13)	13.13 [c] (4.46–38.66)	
Group 4 (n = 29)	12.68 [c] (5.57–28.85)	
Group 5 (n = 182)	4.79 [c] (2.85–8.04)	
# large-scale projects (N2=804)		<0.0001 [c]
Group 1 (n = 11)	67.46(22.42–203.01) [c]	
Group 2 (n = 3)	— [f]	
Group 3 (n = 13)	54.60(19.74–151.03) [c]	
Group 4 (n = 29)	33.03(12.33–88.47) [c]	
Group 5 (n = 182)	21.88(8.64–55.41) [c]	
% challenging research (N=804)		—
Group 1 (n = 11)	0.28(0.00–33.23)	
Group 2 (n = 3)	0.14(0.00–223.48)	
Group 3 (n = 13)	0.93(0.16–5.59)	
Group 4 (n = 29)	1.17(0.42–3.28)	
Group 5 (n = 182)	1.05(0.63–1.73)	
SNIP_first_0–12 y (N=804)		—
Group 1 (n = 11)	1.15(0.44–3.04)	
Group 2 (n = 3)	1.02(0.21–4.88)	
Group 3 (n = 13)	1.34(0.90–2.00)	
Group 4 (n = 29)	1.20(0.82–1.76)	
Group 5 (n = 182)	1.23 [g] (1.02–1.50)	
SNIP_first_13-24 y (N=804)		—
Group 1 (n = 11)	2.95(0.96–9.00)	

	Group 2 (n = 3)	0.00(0.00–0.00)	
	Group 3 (n = 13)	2.28(0.88–5.91)	
	Group 4 (n = 29)	0.05(0.00–2.23)	
	Group 5 (n = 182)	1.28(0.77–2.13)	
SNIP_first_25+ y (N=804)			—
	Group 1 (n = 11)	1.05(0.66–1.67)	
	Group 2 (n = 3)	1.20(0.80–1.78)	
	Group 3 (n = 13)	1.18(0.96–1.45)	
	Group 4 (n = 29)	1.02(0.83–1.26)	
	Group 5 (n = 182)	1.11(1.02–1.21) [e]	
SNIP_second_0–12 y (N=804)			<0.0001 [c]
	Group 1 (n = 11)	2.87 [e] (1.29–6.43)	
	Group 2 (n = 3)	2.66(0.86–8.21)	
	Group 3 (n = 13)	2.66 [c] (1.47–4.82)	
	Group 4 (n = 29)	2.44 [c] (1.52–3.92)	
	Group 5 (n = 182)	2.07 [c] (1.54–2.79)	
SNIP_second_13-24 y (N=804)			—
	Group 1 (n = 11)	1.29(0.68–2.46)	
	Group 2 (n = 3)	0.00(0.00–0.00)	
	Group 3 (n = 13)	1.13(0.61–2.10)	
	Group 4 (n = 29)	1.11(0.72–1.74)	
	Group 5 (n = 182)	1.01(0.79–1.29)	
SNIP_second_25+ y (N=804)			<0.0001 [c]
	Group 1 (n = 11)	2.59 [g] (1.40–4.81)	
	Group 2 (n = 3)	0.66(0.09–5.01)	
	Group 3 (n = 13)	2.28 [c] (1.38–3.77)	
	Group 4 (n = 29)	2.14 [c] (1.44–3.19)	
	Group 5 (n = 182)	1.83 [c] (1.43–2.34)	
SNIP_senior_0–12 y (N=804)			<0.0001 [c]
	Group 1 (n = 11)	3.74(2.08–6.71)	
	Group 2 (n = 3)	2.13 [c] (0.34–13.35)	
	Group 3 (n = 13)	3.58 [c] (2.21–5.81)	
	Group 4 (n = 29)	2.98 [c] (1.86–4.78)	
	Group 5 (n = 182)	2.63 [c] (1.95–3.54)	
SNIP_senior_13-24 y (N=804)			0.023 [g]
	Group 1 (n = 11)	16.96 [e] (2.47–116.26)	
	Group 2 (n = 3)	0.00(0.00–0.00)	

	Group 3 (n = 13)	6.67 [g] (1.43–31.10)	
	Group 4 (n = 29)	1.57(0.31–8.06)	
	Group 5 (n = 182)	1.42(0.66–3.08)	
SNIP_senior_25+ y (N=804)			<0.0001 [c]
	Group 1 (n = 11)	1.81(0.54–6.07)	
	Group 2 (n = 3)	2.98(0.80–11.15)	
	Group 3 (n = 13)	3.27 [c] (1.72–6.21)	
	Group 4 (n = 29)	2.7 [c] (1.69–4.32)	
	Group 5 (n = 182)	1.77 [c] (1.38–2.28)	

[a] The researcher type was used as the dependent variable. Group 6 was used as a reference.

[b] These variables were quantified based on the largest grant category secured by the mentor, with smaller values indicating greater competitiveness.

[c] *P* <.001.

[d] *P*-values greater than 0.05 are represented by em dashes.

[e] *P* <.01.

[f] No results were produced by XLSTAT software for this group.

[g] *P* <.05.
