# Supplementary material for: Individual Research Behaviors and Research Funding Acquisition Across Fields and Career Periods: Regression Analysis
Source: Interact J Med Res. 2026 Jul 27;15:e98428. doi: 10.2196/98428 (PMC13405367; doi:10.2196/98428)
Supplement: Multimedia Appendix 5 [file ijmr-v15-e98428-s005.pdf]

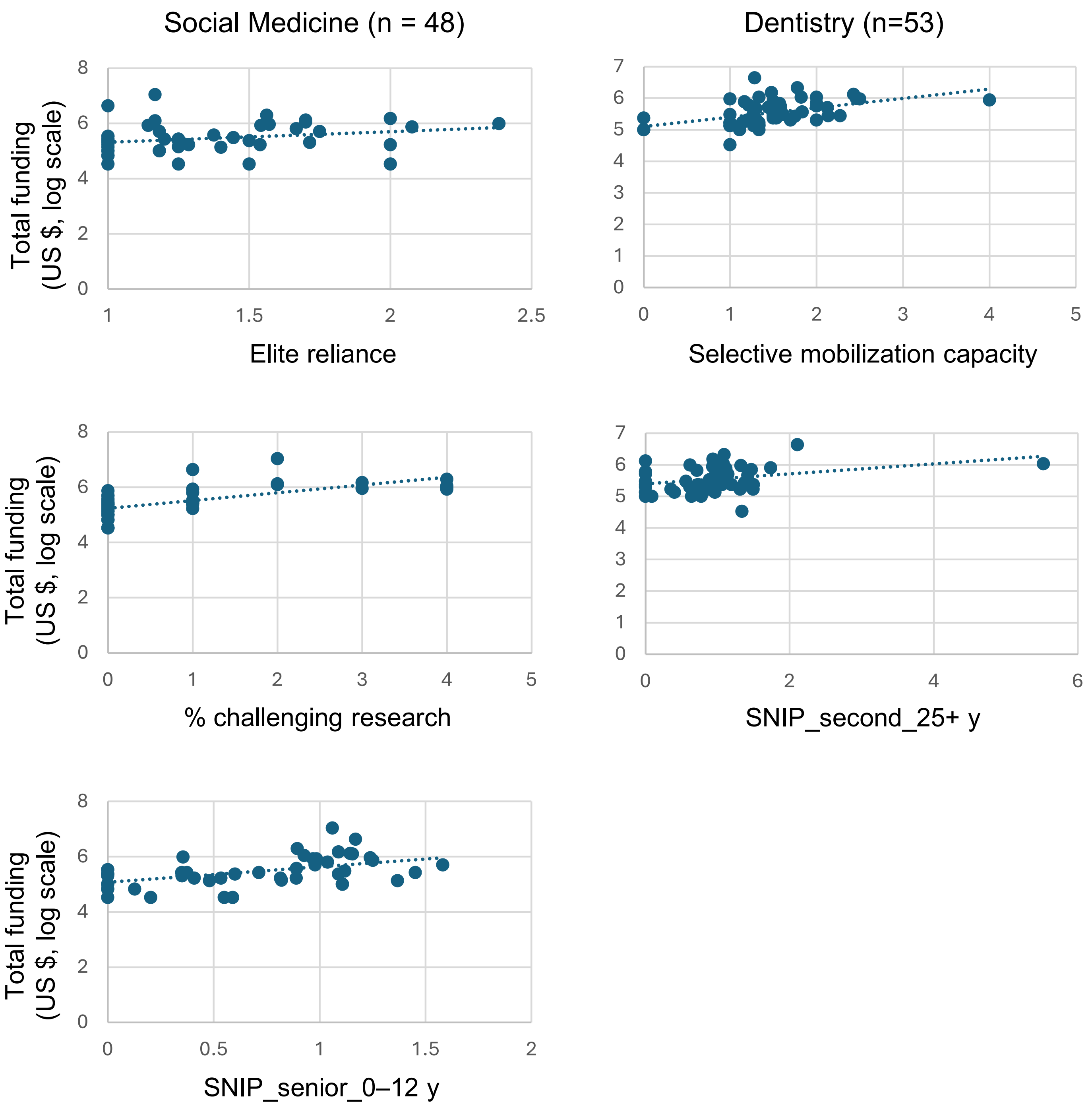

Multimedia Appendix 5. Regression of behavior-related indicators against the amount allocated (log-transformed). A currency exchange rate of JP ¥1=US $0.0067 is applicable.
