# Supplementary material for: Individual Research Behaviors and Research Funding Acquisition Across Fields and Career Periods: Regression Analysis
Source: Interact J Med Res. 2026 Jul 27;15:e98428. doi: 10.2196/98428 (PMC13405367; doi:10.2196/98428)
Supplement: Multimedia Appendix 6 [file ijmr-v15-e98428-s006.pdf]

Multimedia Appendix 6. Correlation matrix (N=804).

Variable	1. Total funding	2. Funding_0–12y	3. Career length	4. University rank (graduated)	5. University rank (currently affiliated)	6. Mentor level	7. # first-authored_0–5y	8. SNIP_0–5y	9. # projects	10. # large-scale projects	11. % challenging research	12. Elite reliance	13. Selective mobilization capacity	14. Total publication counts	15. % first-, second-, and senior-authored publications	16. Average SNIP	17. SNIP_first_0–12 y	18. SNIP_first_13-24 y	19. SNIP_first_25+ y	20. SNIP_second_0–12 y	21. SNIP_second_13-24 y	22. SNIP_second_25+ y	23. SNIP_senior_0–12 y	24. SNIP_senior_13-24 y	25. SNIP_senior_25+ y
**1. Total funding (mean 12319, SD 25117.6; range 1–2305) [c]**																									
r	1.000	0.419	0.170	-0.341	-0.325	-0.341	0.099	0.240	0.847	0.429	0.701	0.142	0.196	0.080	0.142	0.245	0.150	0.048	0.132	0.137	0.059	0.169	0.312	0.103	0.239
P-value	—	<.05	<.05	<.05	<.05	<.05	<.05	<.05	<.05	<.05	<.05	<.05	<.05	<.05	<.05	<.05	<.05	—	<.05	<.05	—	<.05	<.05	<.05	<.05
**2. Funding_0–12y (mean 3168.5, SD 7566.1; range 0–1070) [c]**																									
r	0.419	1.000	-0.120	-0.160	-0.039	-0.175	0.060	0.139	0.439	-0.453	0.255	0.115	0.111	-0.052	-0.041	-0.006	-0.002	-0.141	0.063	-0.041	-0.163	0.123	0.015	-0.147	0.090
P-valu	<.05	—	<.05	<.05	—	<.05	—	<.05	<.05	<.05	<.05	<.05	<.05	—	—	—	—	<.05	—	—	<.05	<.05	—	<.05	<.05

	e																									
**3. Career length (mean 22.8, SD 4.3; range 11–29)**																										
	r	0.170	-0.120	1.000	0.091	0.019	0.051	-0.039	-0.009	0.356	0.074	0.137	0.285	0.217	0.194	0.237	0.151	0.123	0.543	-0.034	0.149	0.627	-0.059	0.164	0.632	-0.046
	P-value	<.05	<.05	—[d]	<.05	—	—	—	—	<.05	<.05	<.05	<.05	<.05	<.05	<.05	<.05	<.05	<.05	—	<.05	<.05	—	<.05	<.05	—
**4. University rank (graduated) (mean 3.2, SD 1.8; range 1–7) [a]**																										
	r	-0.341	-0.160	0.091	1.000	0.420	0.232	0.019	-0.089	-0.244	-0.186	-0.289	0.064	0.045	0.011	0.016	-0.060	-0.024	0.034	0.012	-0.034	0.053	-0.042	-0.099	-0.027	-0.148
	P-value	<.05	<.05	<.05	—	<.05	<.05	—	<.05	<.05	<.05	<.05	—	—	—	—	—	—	—	—	—	—	—	<.05	—	<.05
**5. University rank (currently affiliated) (mean 4.2, SD 1.7; range 1–7) [a]**																										
	r	-0.325	-0.039	0.019	0.420	1.000	0.193	-0.012	-0.050	-0.213	-0.263	-0.274	0.033	-0.023	-0.152	-0.156	-0.178	0.033	-0.011	-0.038	-0.086	-0.007	-0.146	-0.196	-0.068	-0.126
	P-value	<.05	—	—	<.05	—	<.05	—	—	<.05	<.05	<.05	—	—	<.05	<.05	<.05	—	—	—	<.05	—	<.05	<.05	—	<.05
**6. Mentor level (mean 4.7, SD 0.9; range 1–5) [b]**																										
	r	-	-	0.0	0.2	0.1	1.0	-	-	-	-	-	-	0.0	0.0	0.0	-	-	0.0	-	-	0.0	-	-	0.0	-

		0.341	0.175	51	32	93	00	0.008	0.133	0.257	0.162	0.262	0.077	81	74	53	0.044	0.053	57	0.053	0.007	68	0.001	0.033	04	0.137
	P-value	<.05	<.05	0.146	<.05	<.05	—	—	<.05	<.05	<.05	<.05	<.05	<.05	<.05	—	—	—	—	—	—	—	—	—	—	<.05
**7. # first-authored_0–5y (mean 3.6, SD 4.1; range 0–49)**																										
	r	0.099	0.060	-0.039	0.019	-0.012	-0.008	1.000	0.666	0.094	0.086	0.076	0.020	0.046	0.416	0.499	0.493	0.596	-0.044	0.520	0.310	-0.071	0.482	0.275	-0.066	0.303
	P-value	<.05	—	—	—	—	—	—	<.05	<.05	<.05	<.05	—	—	<.05	<.05	<.05	<.05	—	<.05	<.05	<.05	<.05	<.05	—	<.05
**8. SNIP_0–5y (mean 1.5, SD 1.5; range 0–12.2)**																										
	r	0.240	0.139	-0.009	-0.089	-0.050	-0.133	0.666	1.000	0.202	0.109	0.202	0.024	0.021	0.319	0.357	0.457	0.544	-0.069	0.461	0.315	-0.073	0.436	0.310	-0.048	0.303
	P-value	<.05	<.05	—	<.05	—	<.05	<.05	—	<.05	<.05	<.05	—	—	<.05	<.05	<.05	<.05	—	<.05	<.05	<.05	<.05	<.05	—	<.05
**9. # projects (mean 6.6, SD 4.1; range 1–37)**																										
	r	0.847	0.439	0.356	-0.244	-0.213	-0.257	0.094	0.202	1.000	0.294	0.671	0.260	0.307	0.084	0.164	0.214	0.169	0.144	0.118	0.156	0.142	0.157	0.272	0.152	0.136
	P-value	<.05	<.05	<.05	<.05	<.05	<.05	<.05	<.05	—	<.05	<.05	<.05	<.05	<.05	<.05	<.05	<.05	<.05	<.05	<.05	<.05	<.05	<.05	<.05	<.05
**10. # large-scale projects (mean 0.2, SD 0.6; range 0–6)**																										
	r	0.429	-0.453	0.074	-0.186	-0.263	-0.162	0.086	0.109	0.294	1.000	0.320	-0.020	-0.007	0.103	0.146	0.199	0.093	0.045	0.083	0.122	0.074	0.088	0.229	0.096	0.180
	P-value	<.05	<.05	<.05	<.05	<.05	<.05	<.05	<.05	<.05	—	<.05	—	—	<.05	<.05	<.05	<.05	—	<.05	<.05	<.05	<.05	<.05	<.05	<.05
**11. % challenging research (mean 1, SD 1.6; range 0–8)**																										
	r	0.7	0.2	0.1	-	-	-	0.0	0.2	0.6	0.3	1.0	0.1	0.1	0.0	0.0	0.1	0.1	0.0	0.0	0.0	0.0	0.1	0.2	0.0	0.1

		01	55	37	0.289	0.274	0.262	76	02	71	20	00	18	59	31	86	81	21	69	79	88	36	21	53	74	77
	P-value	<.05	<.05	<.05	<.05	<.05	<.05	<.05	<.05	<.05	<.05	—	<.05	<.05	—	<.05	<.05	<.05	<.05	<.05	<.05	—	<.05	<.05	<.05	<.05
**12. Elite reliance (mean 1.5, SD 0.7; range 1–10)**																										
	r	0.142	0.115	0.285	0.064	0.033	-0.077	0.020	0.024	0.260	-0.020	0.118	1.000	0.329	0.103	0.141	0.087	0.064	0.127	0.027	0.113	0.151	0.068	0.089	0.132	-0.084
	P-value	<.05	<.05	<.05	—	—	<.05	—	—	<.05	—	<.05	—	<.05	<.05	<.05	<.05	—	<.05	—	<.05	<.05	—	<.05	<.05	<.05
**13. Selective mobilization capacity (mean 1.3, SD 0.6; range 0–6)**																										
	r	0.196	0.111	0.217	0.045	-0.023	0.081	0.046	0.021	0.307	-0.007	0.159	0.329	1.000	0.090	0.126	0.040	0.023	0.103	0.016	0.044	0.097	0.018	0.087	0.106	-0.034
	P-value	<.05	<.05	<.05	—	—	<.05	—	—	<.05	—	<.05	<.05	—	<.05	<.05	—	—	<.05	—	—	<.05	—	<.05	<.05	0.334
**14. Total publication counts (mean 159.5, SD 129.9; range 1–901)**																										
	r	0.080	-0.052	0.194	0.011	-0.152	0.074	0.416	0.319	0.084	0.103	0.031	0.103	0.090	1.000	0.877	0.810	0.495	0.227	0.433	0.639	0.261	0.593	0.549	0.236	0.275
	P-value	<.05	—	<.05	—	<.05	<.05	<.05	<.05	<.05	<.05	—	<.05	<.05	—	<.05	<.05	<.05	<.05	<.05	<.05	<.05	<.05	<.05	<.05	<.05
**15. % first-, second-, and senior-authored publications (mean 86.2, SD 80.2; range 0–502)**																										
	r	0.142	-0.041	0.237	0.016	-0.156	0.053	0.499	0.357	0.164	0.146	0.086	0.141	0.126	0.877	1.000	0.921	0.573	0.277	0.522	0.676	0.321	0.667	0.629	0.254	0.350
	P-value	<.05	—	<.05	—	<.05	—	<.05	<.05	<.05	<.05	<.05	<.05	<.05	<.05	—	<.05	<.05	<.05	<.05	<.05	<.05	<.05	<.05	<.05	<.05
**16. Average SNIP (mean 68.7, SD 64.5; range 0–470.7)**																										
	r	0.2	-	0.1	-	-	-	0.4	0.4	0.2	0.1	0.1	0.0	0.0	0.8	0.9	1.0	0.6	0.2	0.5	0.7	0.2	0.6	0.6	0.2	0.4

		45	0.006	51	0.060	0.178	0.044	93	57	14	99	81	87	40	10	21	00	63	41	99	27	61	79	50	19	16
	P-value	<.05	—	<.05	—	<.05	—	<.05	<.05	<.05	<.05	<.05	<.05	—	<.05	<.05	—	<.05	<.05	<.05	<.05	<.05	<.05	<.05	<.05	<.05
**17. SNIP_first_0–12 y (mean 8.9, SD 9.4; range 0–63.4)**																										
	r	0.150	-0.002	0.123	-0.024	0.033	-0.053	0.596	0.544	0.169	0.093	0.121	0.064	0.023	0.495	0.573	0.663	1.000	0.255	0.556	0.516	0.172	0.378	0.267	0.102	0.165
	P-value	<.05	—	<.05	—	—	—	<.05	<.05	<.05	<.05	<.05	—	—	<.05	<.05	<.05	—	<.05	<.05	<.05	<.05	<.05	<.05	<.05	<.05
**18. SNIP_first_13-24 y (mean 1, SD 3.1; range 0–45.8)**																										
	r	0.048	-0.141	0.543	0.034	-0.011	0.057	-0.044	-0.069	0.144	0.045	0.069	0.127	0.103	0.227	0.277	0.241	0.255	1.000	0.028	0.186	0.574	-0.039	0.049	0.463	-0.034
	P-value	<.05	—	—	—	—	—	<.05	<.05	<.05	<.05	<.05	—	—	<.05	<.05	<.05	<.05	—	—	<.05	—	<.05	<.05	—	<.05
**19. SNIP_first_25+ y (mean 8.5, SD 9.4; range 0–72.4)**																										
	r	0.132	0.063	-0.034	0.012	-0.038	-0.053	0.520	0.461	0.118	0.083	0.079	0.027	0.016	0.433	0.522	0.599	0.556	0.028	1.000	0.408	-0.040	0.538	0.332	-0.065	0.221
	P-value	—	<.05	<.05	—	—	—	—	—	<.05	—	<.05	<.05	<.05	<.05	<.05	<.05	<.05	—	—	<.05	<.05	—	—	<.05	—
**20. SNIP_second_0–12 y (mean 12.8, SD 14.8; range 0–135.7)**																										
	r	0.137	-0.041	0.149	-0.034	-0.086	-0.007	0.310	0.315	0.156	0.122	0.088	0.113	0.044	0.639	0.676	0.727	0.516	0.186	0.408	1.000	0.287	0.523	0.347	0.122	0.116
	P-value	<.05	—	<.05	—	<.05	—	<.05	<.05	<.05	<.05	<.05	<.05	—	<.05	<.05	<.05	<.05	<.05	<.05	—	<.05	<.05	<.05	<.05	<.05
**21. SNIP_second_13-24 y (mean 2.3, SD 6.6; range 0–99.1)**																										
	r	0.0	-	0.6	0.0	-	0.0	-	-	0.1	0.0	0.0	0.1	0.0	0.2	0.3	0.2	0.1	0.5	-	0.2	1.0	-	0.0	0.5	-

		59	0.163	27	53	0.007	68	0.071	0.073	42	74	36	51	97	61	21	61	72	74	0.040	87	00	0.025	69	01	0.021
	P-value	—	<.05	<.05	—	—	—	<.05	<.05	<.05	<.05	—	<.05	<.05	<.05	<.05	<.05	<.05	<.05	—	<.05	—	—	<.05	<.05	—
**22. SNIP_second_25+ y (mean 11.5, SD 14.6; range 0–110.7)**																										
	r	0.169	0.123	-0.059	-0.042	-0.146	-0.001	0.482	0.436	0.157	0.088	0.121	0.068	0.018	0.593	0.667	0.679	0.378	-0.039	0.538	0.523	-0.025	1.000	0.385	-0.050	0.288
	P-value	<.05	<.05	—	—	<.05	—	<.05	<.05	<.05	<.05	<.05	—	—	<.05	<.05	<.05	<.05	—	<.05	<.05	—	—	<.05	—	<.05
**23. SNIP_senior_0–12 y (mean 15.9, SD 28.2; range 0–245.9)**																										
	r	0.312	0.015	0.164	-0.099	-0.196	-0.033	0.275	0.310	0.272	0.229	0.253	0.089	0.087	0.549	0.629	0.650	0.267	0.049	0.332	0.347	0.069	0.385	1.000	0.100	0.390
	P-value	<.05	—	<.05	<.05	<.05	—	<.05	<.05	<.05	<.05	<.05	<.05	<.05	<.05	<.05	<.05	<.05	—	<.05	<.05	<.05	<.05	—	<.05	<.05
**24. SNIP_senior_13-24 y (mean 2.7, SD 9.5; range 0–120.2)**																										
	r	0.103	-0.147	0.632	-0.027	-0.068	0.004	-0.066	-0.048	0.152	0.096	0.074	0.132	0.106	0.236	0.254	0.219	0.102	0.463	-0.065	0.122	0.501	-0.050	0.100	1.000	0.021
	P-value	<.05	<.05	<.05	—	—	—	—	—	<.05	<.05	<.05	<.05	<.05	<.05	<.05	<.05	<.05	<.05	—	<.05	<.05	—	<.05	—	—
**25. SNIP_senior_25+ y (mean 5.1, SD 15; range 0–190.9)**																										
	r	0.239	0.090	-0.046	-0.148	-0.126	-0.137	0.303	0.303	0.136	0.180	0.177	-0.084	-0.034	0.275	0.350	0.416	0.165	-0.034	0.221	0.116	-0.021	0.288	0.390	0.021	1.000
	P-value	<.05	<.05	—	<.05	<.05	<.05	<.05	<.05	<.05	<.05	<.05	<.05	—	<.05	<.05	<.05	<.05	—	<.05	<.05	—	<.05	<.05	—	—

[a] These variables were quantified based on The Times Higher Education World University Rankings 2023, with smaller values indicating higher ranking.

[b] These variables were quantified based on the size of the GIA projects secured by the mentor, with smaller values indicating greater competitiveness.

[c] The variable was the sum of the amount allocated for each project obtained as principal investigator (million yen, JP ¥).

[d] *P*-values greater than 0.05 are represented by em dashes.
